# Statistical Downscaling over Italy using EQM: CMIP6 Climate Projections for the 1985–2100 Period

**DOI:** 10.1038/s41597-025-05270-8

**Published:** 2025-05-30

**Authors:** G. Fedele, A. Reder, P. Mercogliano

**Affiliations:** CMCC Foundation - Euro-Mediterranean Center on Climate Change, Caserta, Italy

**Keywords:** Environmental sciences, Climate sciences

## Abstract

This study presents SD-EQM_GCMs_IT, a new high-resolution climate projection ensemble dataset over Italy, designed to support climate impact assessments and adaptation strategies. It is derived by statistically downscaling nine CMIP6 General Circulation Models (GCMs) using the Copernicus European Regional ReAnalysis (CERRA) as a training dataset. The Empirical Quantile Mapping (EQM) method ensures a realistic statistical distribution and enhances local climate characterization. The dataset provides daily values at 0.05° resolution from 1985 to 2100 for six Essential Climate Variables (ECVs), including temperature, humidity, wind speed, and precipitation. Two climate scenarios are considered: SSP1-2.6 and SSP3-7.0, for low and high challanges to mitigation and adaptation respectively. This dataset enhances the current climate information available for Italy by bridging the gap between existing CMIP6 global projections and the absence of an ensemble of regional climate models for the same scenarios. By offering high-resolution data, it equips policymakers, industries, and communities with refined climate insights to enhance resilience and adaptation efforts.

## Background & Summary

Climate change is exerting significant pressure across multiple systems, affecting individuals, communities, and nations on personal, social, environmental, and economic levels (EEA, 2024^1^). Understanding how climate will evolve under the latest Shared Socioeconomic Pathways (SSPs) scenarios, which offer a more comprehensive representation of greenhouse gas (GHG) concentration pathways and socioeconomic drivers, is essential for analysing climate change impacts on various systems and supporting informed decision-making and adaptation strategies.

To this aim, current General Circulation Models (GCMs) are fundamental for understanding climate evolution over the 21st century. They are made available through successive phases of the Coupled Model Intercomparison Project (CMIP), with the latest generation released under CMIP6^2^. CMIP6 projections are based on different SSPs, which outline possible global socioeconomic changes up to 2100^3^. These scenarios follow five narratives: sustainable development (SSP1), middle-of-the-road (SSP2), regional rivalry (SSP3), inequality (SSP4), and fossil-fuelled growth (SSP5). They combine new societal development narratives with the previous Representative Concentration Pathways (RCPs), which track changes in atmospheric GHG and aerosol concentrations. Data from CMIP6 models form the foundation of the Intergovernmental Panel on Climate Change’s 6th Assessment Report (IPCC AR6).

Meanwhile, GCMs continuously increase in complexity and accuracy, their current spatial resolution (ranging from ~50 km to several hundred kilometres) still has an overly coarse spatial resolution to accurately reproduce the small-scale variability of temperature, precipitation, and snow cover observed in complex topographic areas^4^. As a matter of fact, the last IPCC (Intergovernmental Panel on Climate Change) Assessment Report (AR) acknowledged that regional climate information for impacts and risk assessment is increasingly robust and mature enough to feed climate services and impacts studies with the higher resolution they need^5,6^. In the context of Italian territory, there is also much evidence of the GCMs scale limits, where large-scale models struggle to capture fine-scale (sub-grid) processes accurately. Generally, local-scale climate assessments require refinement in the horizontal resolution of climate models and the availability of different projections to quantify uncertainty for a clear understanding of confidence levels when providing climate information for impact analyses and adaptation planning. To address this gap, dynamical downscaling (DD) and data-driven methods, including statistical downscaling (SD) and artificial intelligence (AI) techniques, are widely used to obtain high-resolution climate information from large-scale models. Specifically, DD employs a regional climate model (RCM) forced by reanalysis data or GCM outputs to generate detailed climate data for a specific region.

The European branch of the CORDEX initiative (EURO-CORDEX^7^) offers a wide range of climate projections for a fixed domain over Europe up to ~12 km resolution. This multitude of climate projections allows for the use of data in an ensemble configuration (i.e., averaging model outputs), easing the assessment of uncertainty as spread across the ensemble. Albeit cutting-edge data, they are driven by CMIP5 GCMs^8^, an earlier phase of the CMIP initiative. The EURO-CORDEX community is currently advancing this work by incorporating CMIP6 GCMs as forcing^9^; however, these projections are not expected to be released in a short time.

An alternative to improving spatial resolution is the use of Convection-Permitting Regional Climate Models (CP-RCMs) at resolutions lower than 4 km, which explicitly solve the convection sub-grid physical process^10,11^. In Italy, VHR-PRO_IT^10^ is the first and only long climate projection experiment (data from 1981 to 2100) at this scale. However, lacking comparable simulations does not allow for a robust uncertainty assessment, which is crucial for decision-making. As a result, it is primarily regarded as a research-focused product. The absence of additional simulations is largely due to the high computational costs and time required to produce such a long climate data series.

To address these gaps, SD may offer a computationally efficient alternative for providing local-scale climate information. SD establishes statistical relationships between coarse-resolution variables and fine-resolution local data during training, applying these relationships to produce localised climate outputs. Unlike DD, SD methods require fewer computational resources and can be applied across different datasets, identifying relationships that enable accurate downscaling of climate variables from large to local scales. Several SD techniques exist, including physical scaling^12,13^, bias-correction spatial downscaling^14,15^ and artificial neural networks^16–19^. Among these, bias correction, alongside machine learning techniques, is one of the most applied methods due to its computational efficiency, and applicability to multiple climate variables^20,21^. It allows global data to be downscaled to a finer resolution while also correcting potential biases, as it requires a reference dataset for training, often based on observations or gridded reanalysis data. This approach makes the data readily usable for local-scale impact analyses. Results indicate that bias correction methods perform well but depend on high-quality predictors with resolutions close to the target. Perfect prognosis methods, in contrast, are highly sensitive to predictor selection and model structure, often struggling with interannual and spatial variability, which RCMs help address. However, RCMs can still exhibit biases due to both the driving GCM and internal model parameterisations^14,22–25^. To reduce these biases, literature widely recommends SD techniques based on bias adjustment of GCM-RCM projections^14,15,26–29^.

This study presents SD-EQM_GCMs_IT^30^ (Statistical Downscaling through Empirical Quantile Mapping for an ensemble of Global Climate Models over Italy; 10.25424/cmcc-75s8-a308), a new open-access climate dataset up to 2100 over Italy at ~5.5 km horizontal resolution obtained by statistically downscaling a set of nine CMIP6 GCMs projections (see Table 1) using the Empirical Quantile Mapping (EQM) bias adjustment approach^31,32^. The dataset provides daily climate data for key variables, such as mean, maximum, and minimum temperature, mean surface wind speed, mean relative humidity, and cumulated precipitation. For each CMIP6 GCM, it includes the historical experiment for 1985–2014 and projection experiments for 2015–2100 under two SSP scenarios (SSP1-2.6 and SSP3-7.0). These scenarios were chosen in alignment with the CORDEX community, which prioritises them for the ongoing dynamical downscaling of CMIP6. The training dataset for the SD is the Copernicus European Regional ReAnalysis (CERRA)^33,34^ at ~5.5 km resolution, chosen to achieve the highest possible atmospheric resolution over Italy taking advantage of a reanalysis product provided by the Copernicus Climate Datastore (https://cds.climate.copernicus.eu/). It should be mentioned that CERRA does not allow the reconstruction of the processes at the CP scale, but it constitutes the best open-access dataset currently available, which assimilates observational data.

**Table 1:** CMIP6 GCMs ensemble used for SD.

CMIP6 GCM	ECS (°C)	Historical	SSP1-2.6	SSP3-7.0
CESM2*	5,2	No tasmax, tasmin	No hurs	No hurs
CMCC-CM2-SR5*	3,5	No tasmax, tasmin	No tasmax, tasmin	No tasmax, tasmin
CNRM-ESM2-1	4,7	All	No sfcWind	No sfcWind
EC-Earth3-Veg	4,3	No sfcWind	No sfcWind	No sfcWind
IPSL-CM6A-LR	4,6	All	All	All
MIROC6	2,6	All	All	All
MPI-ESM1-2-HR	3	All	All	All
NorESM2-MM*	2,5	All	All	All
UKESM1-0-LL**	5,4	All	All	All

The respective ECS values are shown. The *symbol indicates raw CMIP6 model using a 365-day calendar. The **symbol indicates raw CMIP6 model using a 360-day calendar. CMIP6 GCMs availability of the ECVs: temperature (daily minimum –*tasmin*-, maximum –*tasmax*-, and mean –*tas*-), daily cumulated precipitation –*pr*-, daily mean wind speed -*sfcWind*- and daily mean relative humidity -*hurs*.

SD based on bias-adjustment techniques has been widely applied in the literature across various regions^15,27–29^. However, this is the first time such an approach has been used to generate high-resolution (~5.5 km) climate data for Italy starting from CMIP6 GCMs. Although other statistically downscaled datasets at the global scale exist using similar methods to the SD-EQM applied here^6^, none of them reaches this level of spatial detail.

SD-EQM_GCMs_IT^30^ dataset allows users to access the first complete set of high-resolution projections over Italy based on the new CMIP6 GCMs, providing detailed climate data for an ensemble of models (up to 9 for each variable) and two different scenarios (SSP1-2.6 and SSP3-7.0), thus allowing to address uncertainty for each Essential Climate Variable (ECV). Six variables have been derived: mean, maximum and minimum 2m- temperature, mean 2m- relative humidity, mean surface wind speed and cumulated precipitation (*tas, tasmax, tasmin, hurs, sfcWind* and *pr* respectively). This dataset is expected to enhance hazard and impact assessments (high resolution and uncertainty estimation are two key elements for these applications), by integrating CMIP6 scenarios with existing CMIP5 data, helping to align adaptation policies with the latest scientific insights. The improved spatial and temporal resolution of CMIP6 models is anticipated to provide a more accurate representation of local climate conditions and variability, leading to more complete assessments and informed decision-making.

## Methods

### Input data

The set of CMIP6 GCMs selected for SD is listed in Table 1. It has been chosen following the recommendations of the CORDEX community to cover the full range of projection uncertainties. Table 1 provides the Equilibrium Climate Sensitivity (ECS) values for these GCMs, representing the expected long-term global temperature increase resulting from CO2 doubling. Historical and projection data for SSP1-2.6 and SSP3-7.0 scenarios are available for each GCM (Table 1) and cover 115 years (1985–2100), ensuring consistency across selected models. Table 1 also outlines the available experiments for each ECV, with data accessible through the Earth System Grid Federation (ESGF; https://esgf.llnl.gov) and Copernicus Climate Datastore (https://cds.climate.copernicus.eu/). Only GCMs with both historical and projection experiments available on these platforms were downscaled.

The training dataset for the SD is CERRA^33,34^, a regional reanalysis providing spatially and temporally consistent sub-daily historical reconstructions of atmospheric and surface meteorological variables for Europe at 5.5 km x 5.5 km from 1984 to the present day, using ERA5^34,35^ as lateral boundary conditions. CERRA has been produced using the HARMONIE-ALADIN limited-area numerical weather prediction, and data assimilation system. Although the latter can resolve data holes, the much sparser observational networks in the past periods can impact the quality of analyses leading to less accurate estimates.

### Statistical downscaling workflow

**Fig. 1:**
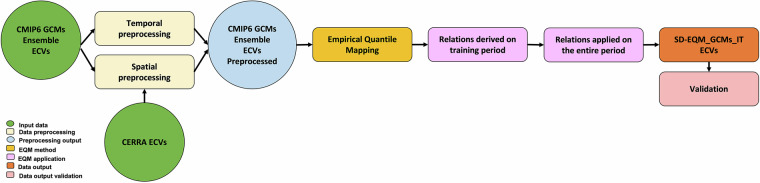
Workflow of the implemented methodology.

Figure 1 shows the schematic workflow of the SD method adopted in this study. It consists of the following main steps: (i) pre-processing all the models to be time-consistent (time preprocessing) with the Gregorian proleptic calendar; (ii) interpolation of CMIP6-GCMs on the CERRA grid (spatial preprocessing) and finally (iii) application of EQM to the CMIP6-GCMs pre-processed in time and space.

Regarding step (i), Table 1 displays which raw models are not time-consistent with this assumption. More specifically, CESM, CMCC-CM2-SR5 and NorESM2-MM use a 365-day calendar. To convert into the proleptic Gregorian calendar, model data have been pre-processed by filling leap-day gaps with averages of the values of February 28th and March 1st of the respective year for *tas, tasmax, tasmin, hurs* and *sfcWind* and with zero values for *pr*. Conversily, UKESM-0-LL is characterised by a 360-day calendar. They are converted into the proleptic Gregorian calendar, pre-processing the model data by inserting the additional missing five days into the raw data after the first 36, 108, 180, 252 and 324 days of each 360-day year and filling with averages of the values of the respective preceding and following days for all variables except for *pr* where values are settled to zero.

**Fig. 2:**
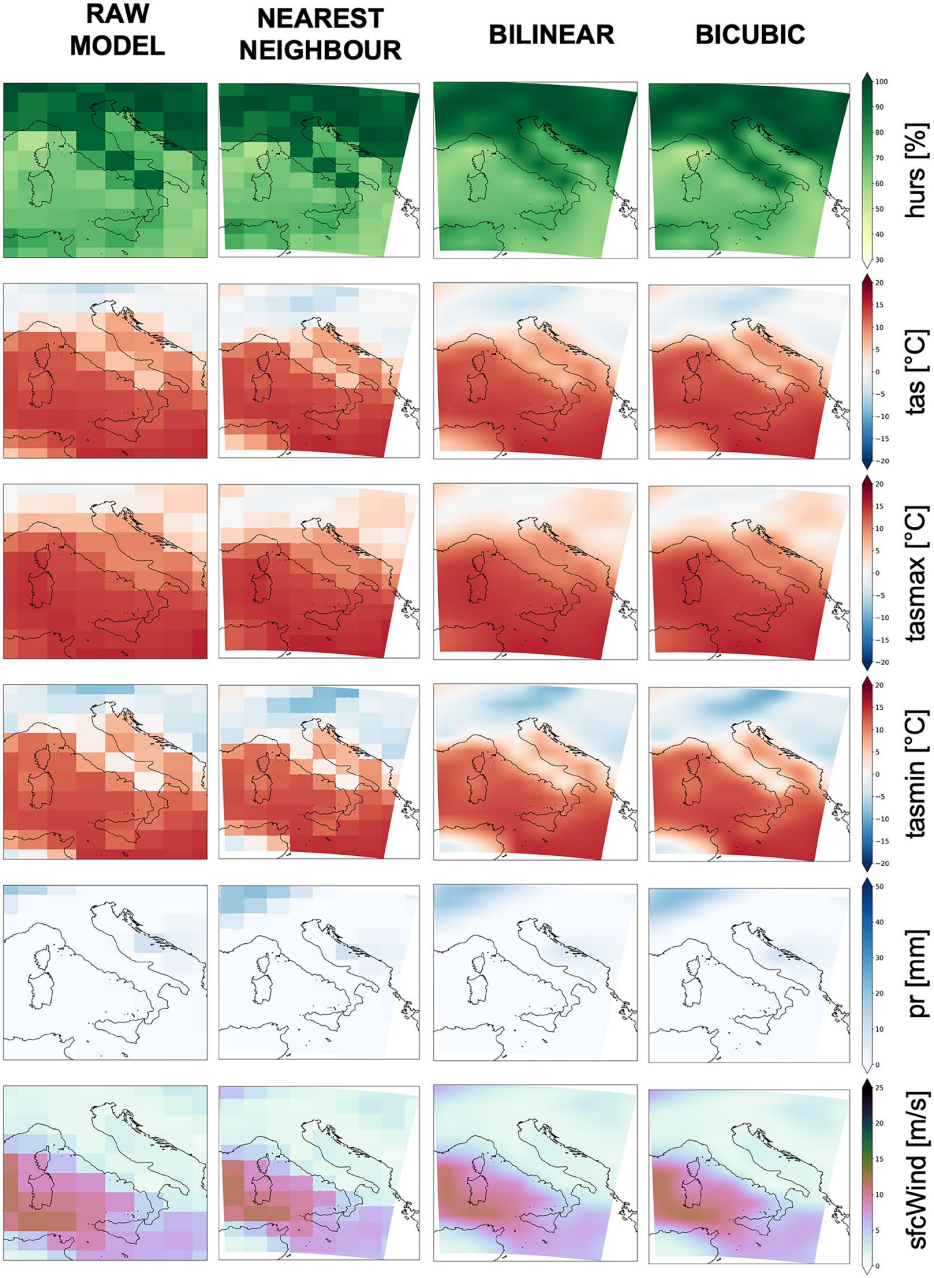
Comparison among raw fields and those obtained through different interpolation methods (nearest neighbour, bilinear and bicubic) for each ECVs. A model (UKESM1-0-LL) containing all the variables has been used focusing on a single timestep (1st of January 1985).

For step (ii), a preliminary investigation of the interpolation method has been performed (Fig. 2), highlighting the gain in adopting a bicubic interpolation. Figure 2 illustrates the impact of the interpolation method adopted on a single timestep (1st of January 1985), supporting as better techniques the bilinear and bicubic methods as they maintain the broadscale patterns relying on the raw model in contrast with the nearest neighbour approach which is impacted by the raw resolution signal. To be consistent with recent applications^15^, the bicubic method has been adopted.

Finally, in step (iii), the bias adjustment technique applied is the Empirical Quantile Mapping. This technique consists of deriving a transfer relation from the cumulative distribution functions (CDFs) of the reference and modelled data^14,36^ to adjust the simulated variables on the observed ones properly. The statistical relationship turning the distribution functions of the modelled variables into the reference ones is:

1 $${x}_{r}=h({x}_{m})={F}_{r}^{-1}[{F}_{m}({x}_{m})]={x}_{m}^{* }$$

where:

$${x}_{r}$$: reference values,

$${x}_{m}$$: model values,

${x}_{m}^{* }$: bias-adjusted values,

$$h$$: transformation function,

$${F}_{r}$$: CDF of $${x}_{r}$$,

$${F}_{m}$$: CDF of $${x}_{m}$$.

Operationally, EQM first estimates the CDFs of both the reference and model data using daily data at regularly spaced quantile levels, employing linear interpolation for values between quantiles. To handle out-of-sample values, it applies constant extrapolation for data below and above the calibration range. The resulting empirical correction function, $h$, is then applied to the projection data, producing bias-adjusted model outputs. To preserve seasonal variability, h is calibrated monthly. For precipitation, EQM aligns wet-day occurrences by adjusting values above a 1 mm/day threshold, ensuring consistency between the reference and model wet/dry day frequencies. Additionally, a frequency adaptation method corrects instances where the model overestimates dry days, allowing for more realistic precipitation simulation.

In the SD procedure, the EQM has been applied point-by-point on a monthly basis to preserve the seasonal patterns and the temporal sequences of climate data. Then, the same relations derived over the training period (1985–2014) have been applied to derive high-resolution climate variables for the entire period of interest (1985–2100), where the timeframe 2015–2100 is assumed as the prediction period.

### Potential limitations

Several sources of uncertainty are associated with the adopted methodology. The first key aspect is the necessity of a long reference time series for model calibration. According to the literature, a minimum of 30 years is required to minimise the influence of internal climate variability. This criterion has been met by using a 30-year calibration period from 1985 to 2014.

A second limitation stems from the assumption of stationarity, which is inherent in the Empirical Quantile Mapping (EQM) method. Casanueva *et al*.^36^ emphasised that bias correction methods introduce considerable uncertainty, as they can alter the climate signal of raw model outputs during the adjustment process. In particular, EQM does not explicitly preserve trends and may modify the climate signal. When EQM fails to preserve the raw model’s trends, impact assessments may be impacted (e.g., over- or under-estimate the magnitude and frequency of future extremes). For example, dampened warming signals lead to lower projections of heatwaves and cold snaps, increasing the risk that health‐risk and agricultural models will underprepare for true conditions. Similarly, altered precipitation trends can misrepresent both flood and drought risks, potentially undermining water‐resource planning and the design of resilient infrastructure. Because extreme events depend on accurate tail behavior, any modification of the distribution’s extremes can skew assessments of ecosystem stress, infrastructure vulnerability and insurance losses. Furthermore, when temperature, humidity and wind trends are not jointly preserved, compound‐event metrics, such as heat‐humidity indices or wind‐driven fire risk, become unreliable. Altogether, these distortions can lead to maladaptation: decision‐makers may under- or over-invest in measures like flood defenses, crop‐management strategies or public health interventions, eroding trust in climate services and reducing the credibility of adaptation planning.

However, this is not necessarily a drawback. Some studies suggest that EQM enhances the climate signal by correcting intensity-dependent errors, especially in cases where absolute threshold-based indices are highly biased and raw signals are unreliable. Others advocate for approaches that maintain relative changes between adjusted and unadjusted data, arguing that fundamental model errors cannot be corrected through statistical adjustments. Trend-preserving techniques are generally preferred unless a modification is physically justified and improves the model’s credibility. A practical way to address this issue is to use an ensemble of bias correction methods to account for projection variability. Nevertheless, since this dataset consists of nine statistically downscaled bias-adjusted models, it inherently accounts for some of the uncertainties introduced by EQM. Additionally, time series for each essential climate variable (ECV) are analysed (see the technical validation section) to assess whether trends are adequately preserved in this application.

The third limitation relates to uncertainties in bias-correction-based spatial downscaling. SD methods often introduce statistical artefacts and may misrepresent spatiotemporal structures. Two key sources of uncertainty are: (i) observational uncertainty and (ii) resolution mismatches between model outputs and reference data. To minimise these uncertainties, ensemble members with varying resolutions have been selected. However, only a single reference dataset has been used. Future work could extend this approach by incorporating multiple observational datasets, thereby expanding the ensemble of SD CMIP6 GCMs. Another possibility may be using Artificial Intelligence approaches^19^ to take advantage of several observational and reanalysis data and then avoid any dependence related to the reference dataset used.

Despite these limitations, EQM remains one of the most widely used methods in the literature due to its advantages: when applied non-parametrically, EQM offers significant flexibility by eliminating the need to select specific statistical functions to model empirical cumulative distribution functions (CDFs)^21^; this adaptability allows EQM to be applied to any climate variable, provided that its distribution can be estimated, regardless of the statistical function that best describes it.

## Data Records

The dataset is available via the CMCC Data Delivery System (https://dds.cmcc.it/#/dataset/cmip6-stat-downscaled-over-italy/; 10.25424/cmcc-75s8-a308)^30^. Each variable, experiment, model and the nomenclature follow the parameterisation below:

*{variable}Adjust_cmip6-stat-downscaled-over-italy_{variable}-{experiment}_{model}_{number}.nc*where:

*variables:* tasmax, tasmin, tas, hurs, sfcWind and pr;

*experiment*: hist, future_ssp126, future_ssp370.

*number* is the download reference number

A couple of examples are shown below:

*prAdjust_cmip6-stat-downscaled-over-italy_pr-future_ssp126_MIROC6_XXXXX.nc*

*prAdjust_cmip6-stat-downscaled-over-italy_pr-hist_MIROC6_XXXXX.nc*

All the metadata are included within the dataset, such as the variable details (description, standard_name, long_name, CoordinateAxisType, units) and the global attributes (author, product, institute_d, frequency, domain, bc_method, bc_method_id, bc_observation). Table 2 provides a general overview of the main features of the SD-EQM_GCMs_IT^30^ dataset. As support for the analyses here shown, additional figures are provided in the Annexes accessible through Zenodo (10.5281/zenodo.15048021)^37^.

**Table 2:** Overview of the key characteristics of the SD-EQM_GCMs_IT dataset.

**Dataset title**	SD-EQM_GCMs_IT (Statistical Downscaling through Empirical Quantile Mapping for an ensemble of Global Climate Models over Italy)
**Dataset type**	Gridded
**Dataset owner/provider**	Fondazione Centro Euro-Mediterraneo sui Cambiamenti Climatici (CMCC)
**Dimensions**	time, longitude, latitude, single vertical level
**Data input format**	NetCDF4 (Climate and Forecast (CF) Metadata Convention; http://cfconventions.org/)
**Data spatial structure**	Lambert conformal conical grid
**Temporal coverage**	From 1985 to 2014 for historical experiments From 2015 to 2100 for SSP experiments
**Temporal resolution**	Daily
**Spatial extent (Horizontal coverage)**	Italian Peninsula land-only: longitude: 3.433062e-05 to 359.9999 degrees_east latitude: 30.26522 to 52.32374 degrees_north
**Coordinate System**	WGS84 (EPSG 4326)
**Spatial resolution (Horizontal resolution)**	≈ 5 km × 5 km (irregular/rotated pole grid)
**Vertical coverage**	Surface, 2 or 10 meters from the surface
**Vertical resolution**	Single levels

Additionally, variation maps derived from this dataset are accessible on the Dataclime platform (www.dataclime.com) for key climate indicators. Some of these indicators are also included in the Italian National Climate Change Adaptation Plan (PNACC), enhancing their usability for a wide range of users and stakeholders.

The dataset contains daily bias-adjusted data on the CERRA Lambert conformal conical grid (~5.5 km) with temporal coverage from 01/01/1985 to 31/12/2100 (i.e., 1985–2014 for the historical period and 2015–2100 for the future projections). These data are provided on a single vertical level (since the variables are near-surface) in NetCDF format (dimensions = time, longitude, latitude, single vertical level) using as reference coordinate system the WGS84 (EPSG 4326). Table 3 reports the list of the output variables (with short and long names), measure units, and a description of the meteorological fields.

**Table 3:** Description of ECVs.

Long-name	Short-name	Units	Description
2 m temperature	tas	°C	Temperature of air at 2 m above the surface
2 m maximum temperature	tasmax	°C	Maximum temperature of the air at 2 m above the surface
2 m minimum temperature	tasmin	°C	Minimum temperature of the air at 2 m above the surface
2 m relative humidity	hurs	%	Relative humidity of the air at 2 m above the surface
Total precipitation amount	pr	mm	cumulated liquid and frozen water, comprising rain and snow, that falls to the surface
Near-surface wind speed	sfcWind	m s^−1^	The magnitude of the two-dimensional horizontal air velocity at 10 m above the surface.

## Technical Validation

This section verifies the adopted statistical downscaling technique by assessing the robustness of the dataset under examination and describes the dataset from the ensemble mean and standard deviation (*std*) perspective, both over the historical and projection periods. Several aspects of the methodology are inspected to emphasise the effects of the statistical downscaling methodology here implemented on the raw GCMs. First, we evaluate whether the climatology of each SD-EQM GCM is properly bias-adjusted with respect to the reference dataset, focusing the analyses on the training period. Next, the effects induced by the implemented technique on the raw models’ variability and trend are discussed and finally, the ECVs ensemble mean and standard deviation are presented.

Given the large number of selected models and their combinations with ECVs across two scenarios, except for some figures, only a few examples are shown to enhance the readability of the paper. However, for completeness, figures corresponding to each combination are provided in the Annexes accessible through Zenodo (10.5281/zenodo.15048021)^37^.

### Climatology evaluation over the training period

**Fig. 3:**
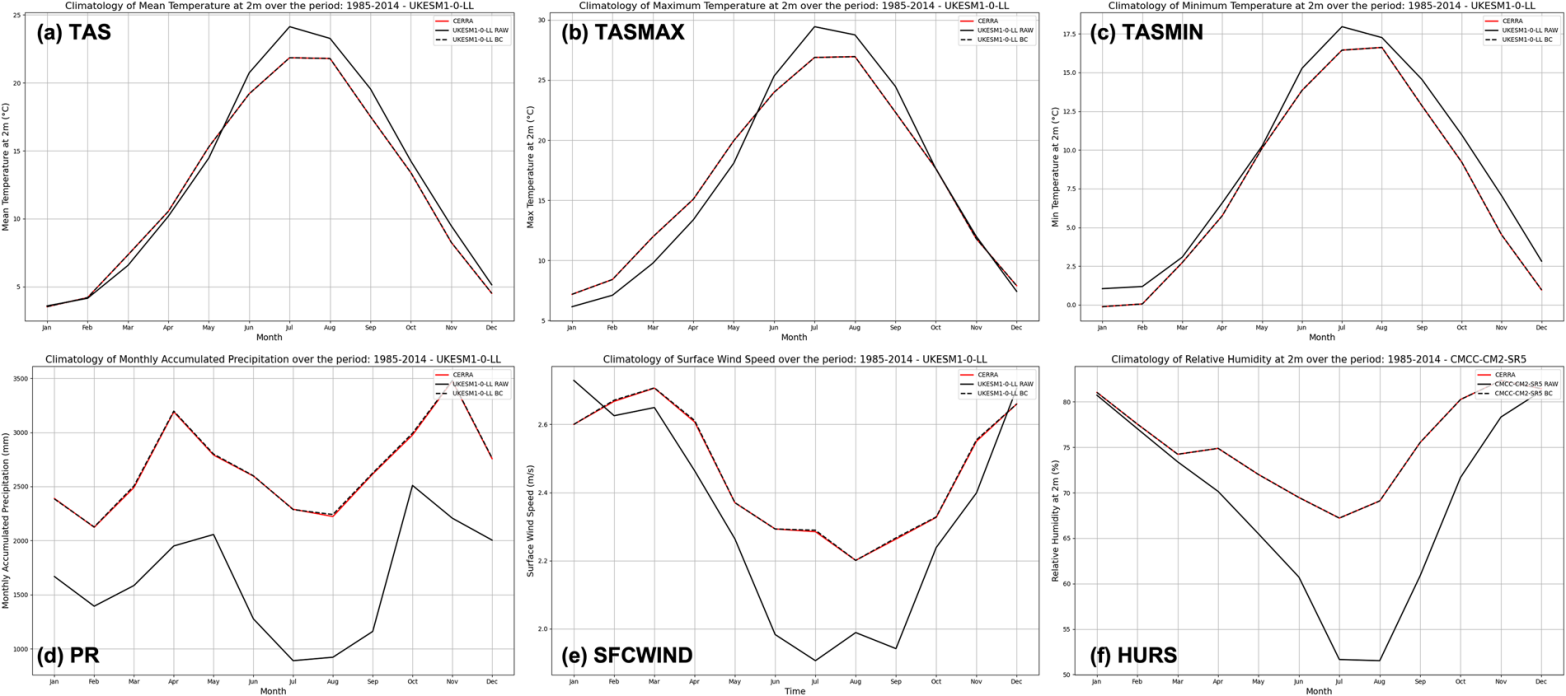
Climatology for the UKESM1-0-LL model for daily ECVs before (RAW, tick black line) and after (BC, dashed black line) bias-correction against the reference climatology provided by CERRA (red line) over the calibration period: 1985–2014. The ECVs’ climatology is presented as follows: (**a**) Monthly Mean of Mean Daily 2m-Temperature, (**b**) Monthly Mean of Maximum Daily 2m-Temperature, (**c**) Monthly Mean of Minimum Daily 2m-Temperature, (**d**) Multi-year monthly sum precipitation, (**e**) Monthly Mean of Mean Daily Surface Wind Speed, (**f**) Monthly Mean of Mean Daily Relative Humidity.

The statistical downscaling methodology here adopted relies on the assumption that the climatology of the reference dataset should be properly reconstructed. This is shown in Fig. 3, which illustrates the climatology for a single GCM (UKESM1-0-LL, randomly chosen among models with all available variables) to highlight the successful correction of climatological bias following the application of EQM to each variable (other models presented in Annex 1). The monthly means over the training period (1985–2014) are presented, except for precipitation, where the multi-year monthly sum over the 30-year period is preferred. This comparison is conducted between the raw GCMs and the bias-corrected fields to properly assess the improvements introduced by the SD-EQM relative to the original dataset. Consequently, no comparison is made with the intermediate step involving the remapped variables. Figure 3 highlights the presence of systematic biases in each ECV within the raw GCMs. However, after bias correction, these biases collapse to zero over the training period when evaluated in terms of climatological signal. As shown, the bias-corrected climatology for each variable closely aligns with the corresponding CERRA curve.

**Fig. 4:**
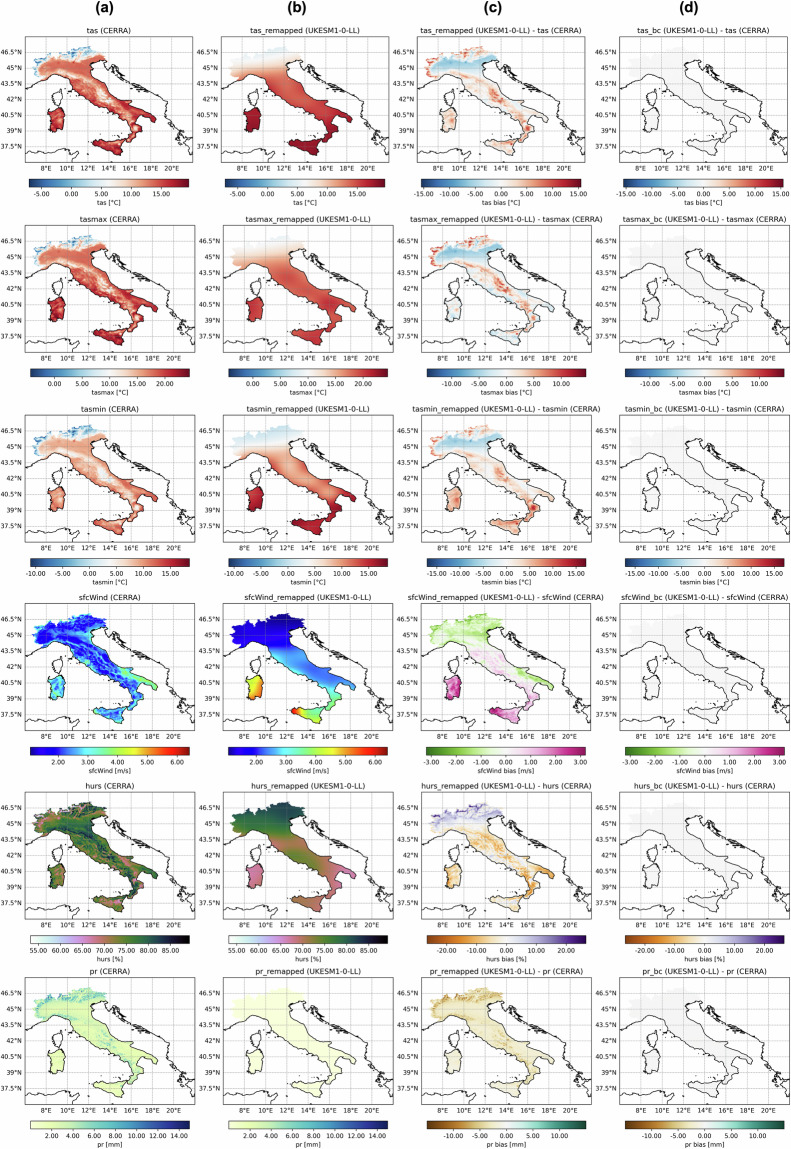
Map of the mean bias for each ECV over the Italian territory. From left to right for each raw it is shown: mean field over calibration period in CERRA, mean field over calibration period in UKESM1-0-LL, mean bias between mean field over remapped model and mean field over CERRA, mean bias between mean field over bias-corrected model and mean field over CERRA.

The bias-removal is also emphasised in Fig. 4, which displays the mean field of each ECV for a single model (see Annex 2 for additional GCMs; 10.5281/zenodo.15048021)^37^ interpolated onto the CERRA grid (referred to as “remapped”), alongside the corresponding CERRA ECV and the mean bias between them, both before and after applying the EQM. Figure 4 illustrates a significant reduction in the mean bias for each investigated variable applying the EQM. By comparing the CERRA variables with the remapped counterparts derived from UKESM1-0-LL (shown here), it is evident that bicubic interpolation on the same grid allows the model to approximate the broad-scale patterns over the Italian Peninsula. However, it still fails to accurately capture local-scale features. The discrepancy between CERRA and the raw variable reaches ~16 °C for *tas, tasmax* and *tasmin*, ~3 m/s for *sfcWind*, ~25% for *hurs* and ~15 mm for *pr*. While, after bias-adjustment all of them converge to zero. Specifically, for temperature variables (tas, tasmax, and tasmin), these discrepancies are particularly pronounced in mountainous regions, such as the Alps and Apennines, where the remapped fields exhibit a systematic positive bias up to ~16 °C compared to CERRA. Similarly, for mean daily wind speed, significant differences are observed: negative biases prevail in Northern Italy and Apulia, while positive biases dominate Central Italy, Campania, Calabria, Sicily, and Sardinia. Notably, the islands exhibit positive biases of up to ~3 m/s. Regarding daily relative humidity at the surface, as with the other variables, local-scale variations are not well captured when interpolating the GCM data onto the CERRA grid. Instead, the intermediate dataset exhibits more uniform patterns with smoother gradients. Widespread negative biases (up to ~−25% with peaks over Sardinia, Calabria and Apulia regions) are found across most of the territory, except for a few localised areas and Northern Italy, where strong positive biases (up to ~25% over the northernmost Alps regions) are observed. For precipitation, negative biases characterise the entire Italian peninsula. Those biases are significantly reduced once the bias correction is applied, with values converging toward zero for each ECV as expected.

### Climate variability and trends

Results shown in the previous section demonstrate strong performance during the training period in reconstructing the reference climatology and mitigating systematic biases. Nonetheless, it remains crucial to assess the impact of applying SD-EQM on climate variability and trends across the entire time series.

**Fig. 5:**
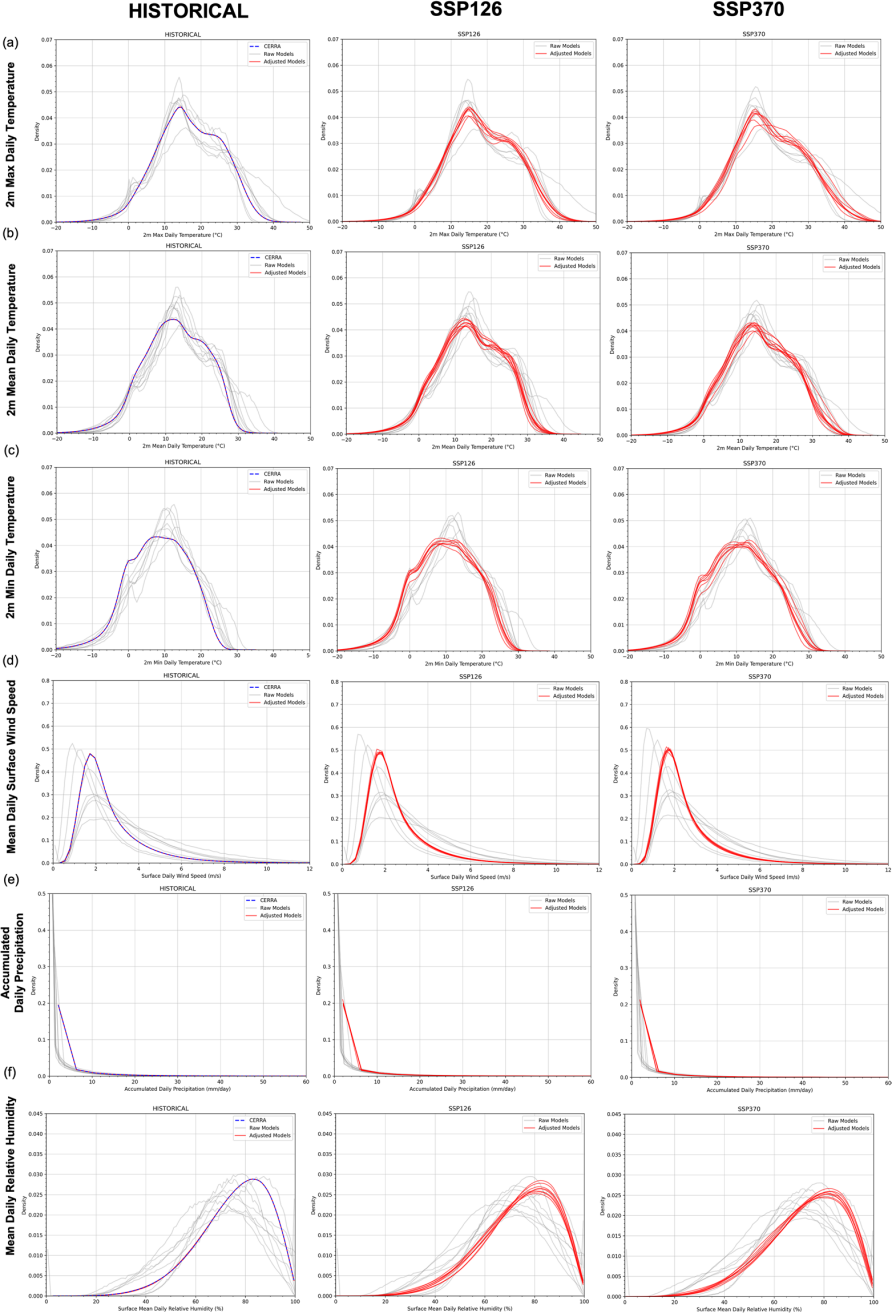
Probability density functions (PDFs) for 2 m (a) maximum, (b) mean, (c) minimum daily temperature, (d) mean surface daily wind speed, (e) cumulated daily precipitation) and 2 m (**f**) mean daily relative humidity averaged over Italy, for the historical, ssp1-2.6 and ssp3-7.0 experiments. Both raw (gray line) and adjusted (red line) ensemble members ECVs are shown. In blue within the historical period’s plots (left), the CERRA pdf averaged over Italy is also included.

**Fig. 6:**
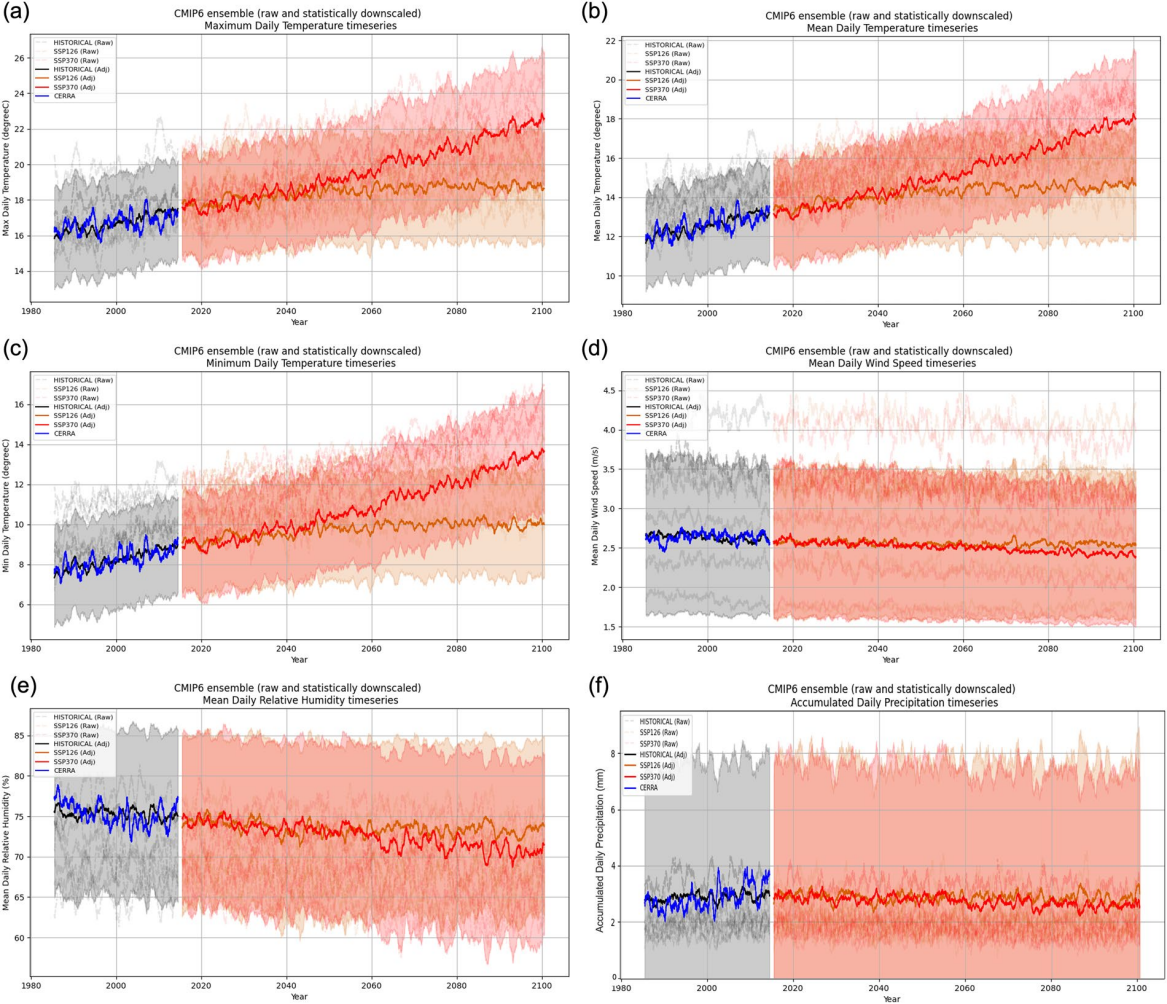
CMIP6 ensemble raw (dashed lines) timeseries for 2 m (**a**) maximum, (**b**) mean, (**c**) minimum daily temperature, (**d**) mean surface daily wind speed, 2 m (**e**) mean daily relative humidity. and (**e**) cumulated daily precipitation. For each subplot the adjusted ensemble mean (dotted line) and spread (colour interval) is shown for each experiment: historical (black), ssp1-2.6 (orange) and ssp3-7.0 (red). The CERRA timeseries is also shown in blue.

This evaluation is important because, by design, SD-EQM may not always preserve climate signals, which is why trend-preserving methods are often recommended. In this context, Casanueva *et al*.^36^ critically examined the impact of eight bias adjustment methods on temperature and precipitation fields over the Iberian Peninsula. Their study highlights the challenges posed by observational uncertainty and resolution mismatches in regional climate applications, emphasising how different bias correction techniques can significantly affect the preservation of climate signals. This issue is particularly relevant when observational datasets contain inherent uncertainties or differ in spatial resolution from climate model outputs. The findings reinforce a key dilemma in the bias correction community: while adjustments improve model realism, they may also distort essential climate trends and variability. Casanueva *et al*.^36^ stress the importance of carefully selecting bias correction methods to maintain the integrity of future climate projections. However, some studies^31,38^ argue that preserving the climate signal is not necessarily essential (as mentioned in the “Potential Limitations” section). Instead, they emphasise that the primary goal of bias correction should be to align model outputs with observational data, thereby improving the realism of future projections. In this regard, Figs. 5, 6 support our evaluation by enabling an investigation of climate variability and climate trends, respectively. Specifically, Fig. 5 presents the probability density functions (PDFs) for the raw ensemble models and the bias-adjusted ones at the daily frequency for each variable over both the historical period and two future scenarios. The historical period also includes CERRA data for comparison. This figure allows us to assess the consistency between raw and adjusted climate variability after applying the SD-EQM technique to both historical data and scenarios. It illustrates the probability density functions (PDFs) for the raw ECVs compared to the adjusted ECVs averaged over Italy. To highlight how the variability of the reference dataset influences changes in the variability of SD-EQM_GCMs_IT^30^ data, the CERRA distribution is also shown for the historical period. For each variable, the impact of the SD-EQM technique on the PDF of the adjusted variable is highlighted. The probability distributions converge toward CERRA’s, both in terms of mean and standard deviation, as quantified in Tables 4, 5, where metrics related to the raw and adjusted ensemble mean are shown. This adjustment affects not only the range of value of the raw model but also the frequencies of its main clusters. The higher the difference between the raw model and CERRA, the stronger the correction applied. Consequently, when the standard deviation of CERRA is lower/higher than that of the raw models, SD-EQM reduces/increases the standard deviation in the adjusted data. This effect extends to the ensemble mean, influencing variability not only in the historical period but also in future projections (Tables 4, 5). A hint of bimodal variability emerges in the historical period and two scenarios in *tasmax* and *tas*: a main mode in the historical period peaking at ~14, 12 °C and a second mode centred around ~26 and 29 °C for *tasmax* and *tas* respectively. Those two peaks are still maintained in both scenarios for each variable, with a shift toward higher values as expected.

**Table 4:** Mean and standard deviation over the entire period for each ensemble mean variable (tasmax, tasmin, tas) for CERRA, raw ensemble mean and adjusted ensemble mean.

	Experiment	Data Type	Mean over the entire period	Standard Deviation over the entire period	Trend per year
**tasmax [degreeC]**	reanalysis	CERRA	16,71	7,35	0,0001
	historical	Raw Ensemble Mean	17,31	8,06	0,0001
	historical	Adjusted Ensemble Mean	16,71	7,03	0,0002
	ssp126	Raw Ensemble Mean	19,36	8,75	0,0000
	ssp126	Adjusted Ensemble Mean	18,46	7,45	0,0000
	ssp370	Raw Ensemble Mean	20,38	9,02	0,0001
	ssp370	Adjusted Ensemble Mean	19,70	7,85	0,0002
tasmin [degreeC]	reanalysis	CERRA	8,22	6,41	0,0001
	historical	Raw Ensemble Mean	10,04	6,45	0,0001
	historical	Adjusted Ensemble Mean	8,22	6,09	0,0002
	ssp126	Raw Ensemble Mean	11,49	6,73	0,0000
	ssp126	Adjusted Ensemble Mean	9,74	6,45	0,0000
	ssp370	Raw Ensemble Mean	12,65	7,09	0,0001
	ssp370	Adjusted Ensemble Mean	10,93	6,85	0,0002
**tas [degreeC]**	reanalysis	CERRA	12,57	6,87	0,0001
	historical	Raw Ensemble Mean	13,72	7,12	0,0001
	historical	Adjusted Ensemble Mean	12,57	6,57	0,0002
	ssp126	Raw Ensemble Mean	15,57	7,51	0,0000
	ssp126	Adjusted Ensemble Mean	14,21	6,94	0,0000
	ssp370	Raw Ensemble Mean	16,39	7,78	0,0001
	ssp370	Adjusted Ensemble Mean	15,33	7,31	0,0002

For each combination above mentioned, trend per year is also provided. Mean, standard deviation and trends over historical, ssp126 and ssp370 refer to the entire period (1985–2014 for the historical period and 2015–2100 for both scenarios).

**Table 5:** Mean and standard deviation over the entire period for each ensemble mean variable (sfcWind, hurs and pr) for CERRA, raw ensemble mean and adjusted ensemble mean.

	Experiment	Data Type	Mean over the entire period	Standard Deviation over the entire period	Trend per year
**sfcWind [m/s]**	reanalysis	CERRA	2,64	0,71	2E-06
	historical	Raw Ensemble Mean	3,16	0,84	−5E-06
	historical	Adjusted Ensemble Mean	2,64	0,39	−9E-06
	ssp126	Raw Ensemble Mean	3,10	1,01	−5E-07
	ssp126	Adjusted Ensemble Mean	2,55	0,39	−1E-06
	ssp370	Raw Ensemble Mean	2,88	0,75	−4E-06
	ssp370	Adjusted Ensemble Mean	2,50	0,38	−6E-06
**hurs [%]**	reanalysis	CERRA	75,32	7,86	−0,0002
	historical	Raw Ensemble Mean	70,95	8,56	−0,0001
	historical	Adjusted Ensemble Mean	75,33	5,42	0,0000
	ssp126	Raw Ensemble Mean	69,45	9,42	0,0000
	ssp126	Adjusted Ensemble Mean	73,53	6,42	0,0000
	ssp370	Raw Ensemble Mean	68,62	10,11	−0,0001
	ssp370	Adjusted Ensemble Mean	72,56	7,26	−0,0001
**pr [mm]**	reanalysis	CERRA	2,86	3,39	8E-05
	historical	Raw Ensemble Mean	2,12	1,46	7E-06
	historical	Adjusted Ensemble Mean	2,88	1,39	2E-05
	ssp126	Raw Ensemble Mean	2,00	1,51	−7E-07
	ssp126	Adjusted Ensemble Mean	2,87	1,49	−1E-06
	ssp370	Raw Ensemble Mean	1,94	1,48	−4E-06
	ssp370	Adjusted Ensemble Mean	2,74	1,50	−1E-05

For each combination above mentioned, trend per year is also provided. Mean, standard deviation and trends over historical, ssp126 and ssp370 refer to the entire period (1985–2014 for the historical period and 2015–2100 for both scenarios).

Larger differences between raw and adjusted variability are more pronounced for variables whose distributions differ significantly between CERRA and the original GCMs. Notably, mean daily surface wind speed and mean daily relative humidity exhibit the most significant changes due to their distinct distributions. Changes in mean and spread for the historical, SSP1-2.6, and SSP3-7.0 scenarios are summarised in Tables 4, 5 for *tas, tasmax, tasmin* and *sfcWind, hurs* and *pr* respectively. Our results are consistent with those found in other similar studies such as Dosio and Paruolo (2011)^14^.

**Fig. 7:**
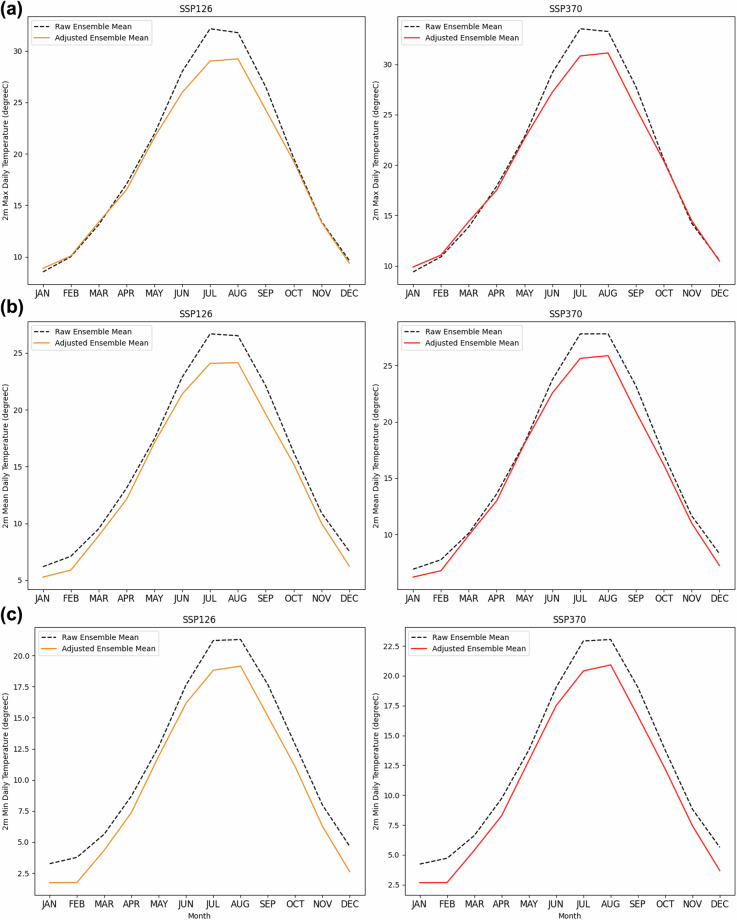
Ensemble mean climatology over future scenarios ssp1-2.6 (left) and ssp3-7.0 (right) for (**a**) 2 m maximum, (**b**) mean and (**c**) minimum daily temperature. In dashed black the raw ensemble mean for each variable and experiment, in orange the adjusted ensemble mean for ssp1-2.6 (2015–2100) and in red the adjusted ensemble mean for ssp3-7.0 (2015–2100).

**Fig. 8:**
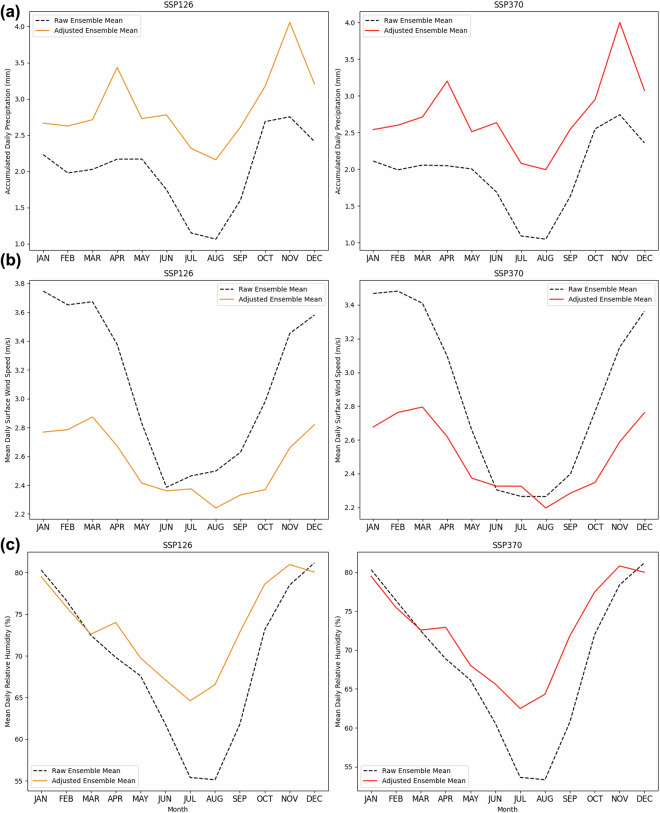
Ensemble mean climatology over future scenarios ssp1-2.6 (left) and ssp3-7.0 (right) for (**a**) cumulated daily precipitation, (**b**) mean daily surface wind speed and (**c**) mean daily relative humidity. In dashed black the raw ensemble mean for each variable and experiment, in orange the adjusted ensemble mean for ssp1-2.6 (2015–2100) and in red the adjusted ensemble mean for ssp3-7.0 (2015-2100).

The effects of SD-EQM on trends are shown in Fig. 6, which presents the yearly running mean timeseries of the investigated daily variables both for CERRA and the raw and adjusted models. This approach maintains the daily resolution while emphasising multiannual variability. The raw CMIP6 ensemble mean over each experiment is also shown for completeness. By visual inspection, it is highlighted by Fig. 6, that the adjusted historical time series converges toward the reference. Moreover, most of the raw CMIP6 models fall within the mean ± standard deviation range of the bias-adjusted ensemble. However, models that strongly deviate from CERRA experience greater correction, leading to values that are no longer well represented in the adjusted ensemble. Despite these corrections, the overall trends remain consistent with the original models (no significant differences detected; see Tables 4, 5). SD-EQM also influences seasonal patterns in future projections. Figures 7, 8 illustrate the climatological signals for each variable under the SSP1-2.6 and SSP3-7.0 scenarios (2015–2100). As expected, variables exhibiting the greatest historical variability differences from CERRA, such as wind speed, precipitation, and relative humidity (Fig. 5), also show significant differences in their adjusted future climatologies. For *tasmax*, the largest differences are observed from June to August in both scenarios, while they approach zero during the remaining months. A similar pattern is found in summer for *tasmean* and *tasmin*; however, unlike *tasmax*, they also exhibit slight differences throughout the rest of the year. While inspecting *pr*, *sfcWind* and *hurs*, as mentioned above, the discrepancies in signal over the two scenarios are emphasized. This outcome is expected for two reasons. First, the distribution differences between the reference data and the GCMs during the training period were more pronounced for these variables than for temperature, resulting in more significant corrections. Second, these variables are more locally dependent, so improvements in resolution have a greater impact on the native signal. It is worth to mention that those results (Figs. 7, 8) aim to emphasize the role of Empirical Quantile Mapping on the scenarios for each ECV, therefore the non-stationarity of climate would play a crucial role considering the entire 2015–2100 period.

To better preserve the climate signal in projections, alternative methods should be considered, as previously discussed. However, it is worth noting that studies^31,38^ suggest that preserving the climate signal during bias correction is not essential. Instead, the primary goal should be to align model outputs with observational data to enhance the realism of future projections. Since each statistical downscaling method has its own assumptions, advantages, and limitations, it is advisable to use multiple techniques that are computationally efficient and less resource-intensive, reducing reliance on a single approach. In this context, machine learning methods^19^ could provide an alternative to reference-dependent techniques like SD-EQM, which significantly modify the GCMs’ native mean and standard deviation to align more closely with reference values.

### Ensemble mean and spread analysis

This section aims to describe the statistical downscaled variables, presenting them from the ensemble mean and standard deviation perspective.

**Fig. 9:**
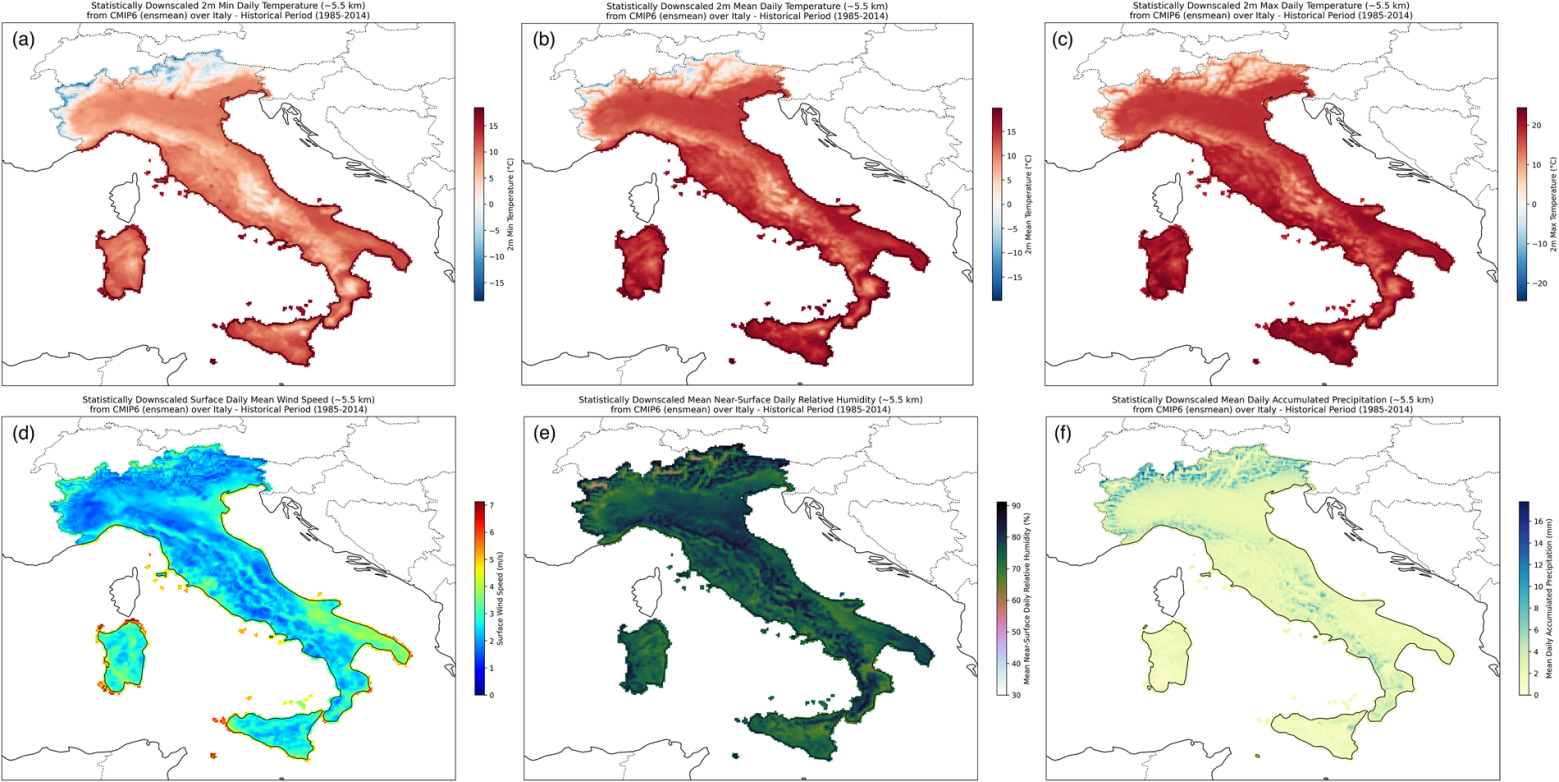
Ensemble mean map over the historical period (1985–2014) for 2 m (**a**) minimum, (**b**) mean, (**c**) maximum daily temperature, (**d**) mean surface daily wind speed, (**e**) 2 m mean daily relative humidity and (**e**) cumulated daily precipitation.

The ensemble mean map for each variable over the historical period is presented in Fig. 9. As expected, local features are better captured in Fig. 9 when comparing the adjusted variables to both the raw model and the corresponding interpolated field (Fig. 2). Each variable matches the reference one (Fig. 4) over the historical period (same as the training period). Positive temperature (max, min and mean) patterns characterize most of the territory, with higher values along the coasts, the southernmost regions, Sicily and Sardinia. While, negative values are found over the Alps for both maximum, minimum and mean temperature and some more localized regions over the Apennines for *tasmin*. Wind speed presents a pattern, strongly impacted by orography. Higher values characterize lowland areas, such as Emilia Romagna, Apulia regions and along the coasts. Along with the previous variables, the mean daily relative humidity and the accumulated daily precipitation also capture local-scale features, consistent with the CERRA pattern (Fig. 4).

**Fig. 10:**
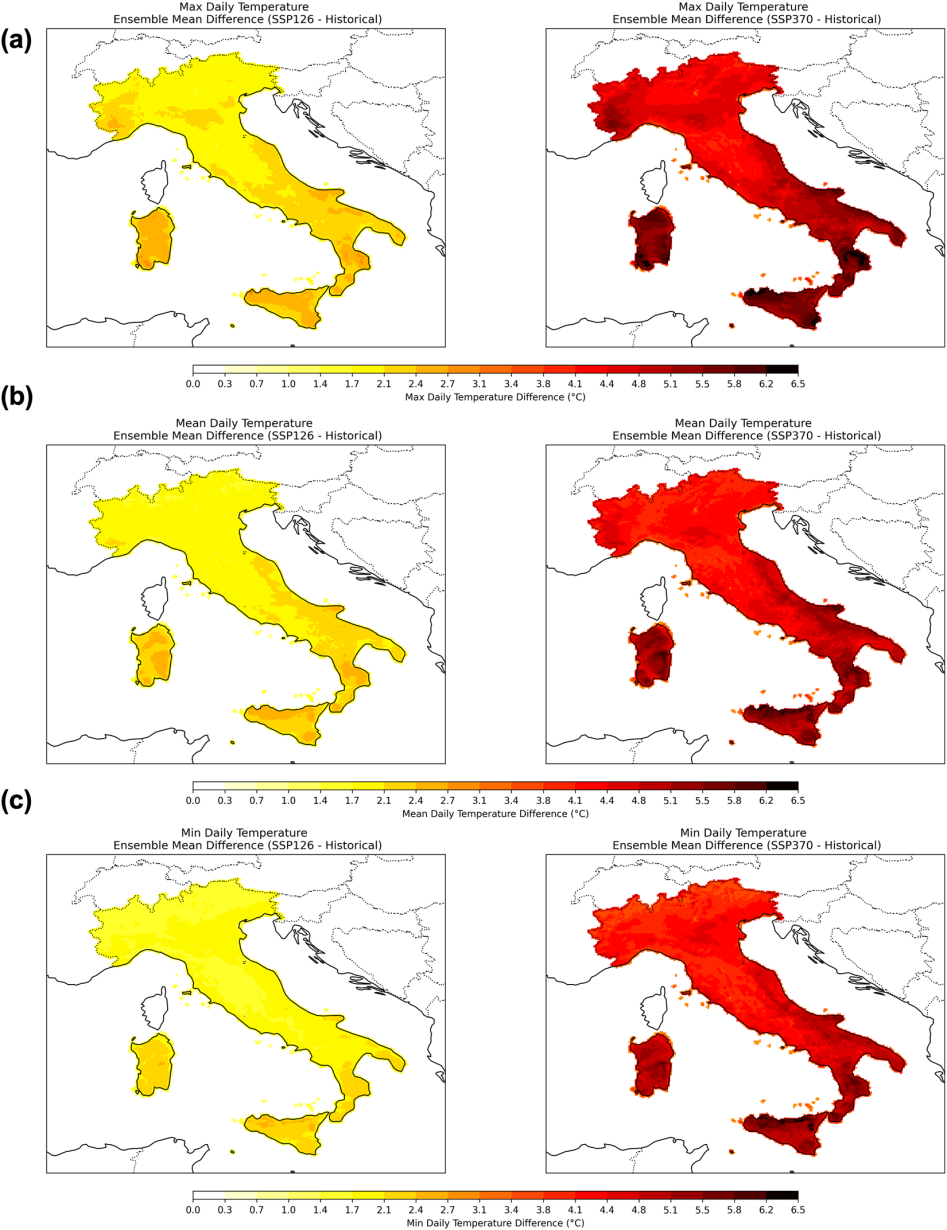
Ensemble mean difference between ssp1-2.6 (left)/ssp3-7.0(right) over the period 2071–2100 mean state and historical mean state (1985–2014) for 2 m daily (**a**) maximum, (**b**) mean and (**c**) minimum temperature.

**Fig. 11:**
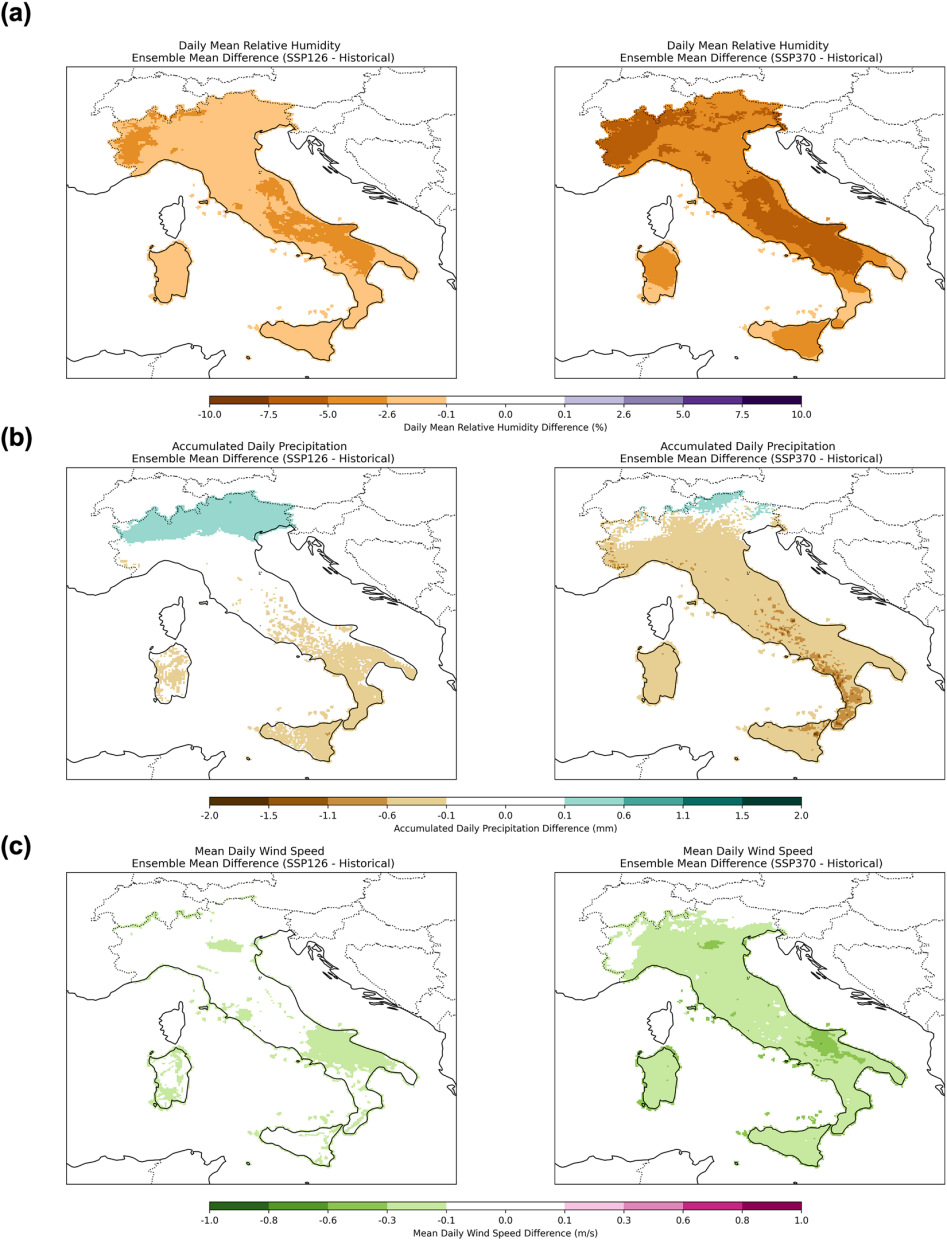
Ensemble mean difference between ssp1-2.6 (left)/ssp3-7.0(right) mean state and historical mean state (1985–2014) for (**a**) 2 m daily relative humidity, (**b**) cumulated precipitation and (**c**) surface daily mean wind speed.

Figures 10, 11 present the ensemble mean differences for each variable by comparing the future climate, represented by projections under SSP1-2.6 and SSP3-7.0 for the period 1971–2100 to facilitate a 30-year climate comparison, with the historical climate spanning 1985–2014. Daily temperatures (maximum, mean, minimum) are presented in Fig. 10, while mean daily relative humidity, daily cumulated precipitation, and mean daily wind speed are shown in Fig. 11. A temperature increase (mean, maximum, and minimum) over the entire territory is shown in Fig. 10, with differences reaching up to ~2,7 °C across the entire territory under SSP1-2.6, with higher values observed in Sardinia, Sicily, Calabria, and Apulia (with *tasmax* also peaking in Liguria). A similar spatial pattern emerges in SSP3-7.0, though with even higher temperature increases, reaching up to ~6,5 °C. Liguria, Piemonte and Southern Italy are the regions with stronger temperature increases expected in both scenarios.

For relative humidity (Fig. 11a), a general decrease in mean daily values is captured, with decreases down to ~5% under SSP1-2.6 and ~7,5% under SSP3-7.0. The most pronounced reductions occur in northwestern and central-south Italy for both scenarios.

Unlike temperature and humidity, which show a consistent increasing or decreasing “monopole” trend, cumulated precipitation exhibits a bipolar pattern (Fig. 11b). Specifically, in the scenario SSP1-2.6, northern central and eastern Italy experiences increased precipitation, while no significant changes are expected to impact central Italy. Finally, a decrease in precipitation characterises southern Italy, Sicily and the innermost sector of the Sardinia region. Under the SSP3-7.0 scenario, a different pattern emerges: northeastern Italy shows localised positive differences, whereas most of the country experiences a negative trend, with Campania and Calabria registering the largest declines.

In addition, wind speed decreases under both scenarios, as illustrated in Fig. 11c. For SSP3-7.0, significant reductions occur over most of Italy, while for SSP1-2.6, only a few localised regions are projected to experience a decrease in mean daily surface wind speed.

**Fig. 12:**
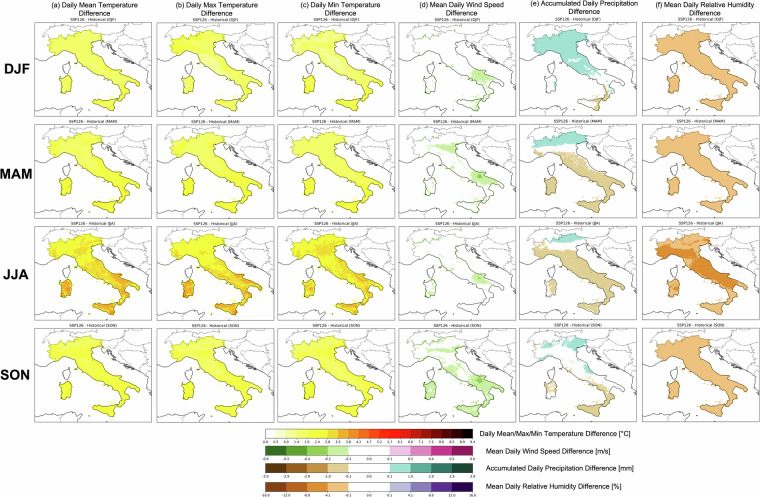
Ensemble mean difference for each season (DJF, MAM, JJA, SON from top to bottom respectively) between ssp1-2.6 over the period 2071–2100 and historical mean state (1985–2014) for (**a**) 2 m daily relative humidity, (**b**) cumulated precipitation and (**c**) surface mean daily wind speed.

**Fig. 13:**
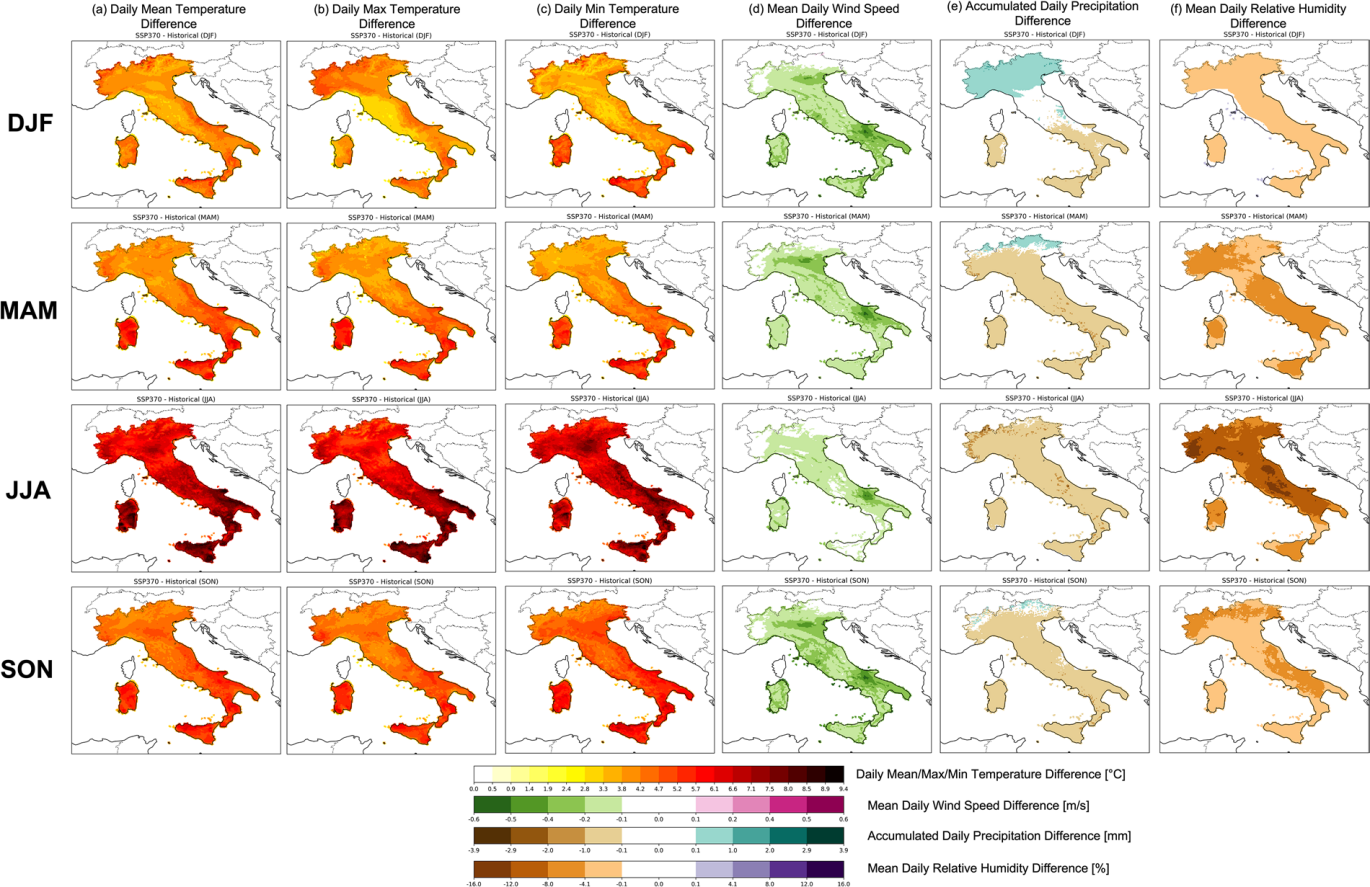
Ensemble mean difference for each season (DJF, MAM, JJA, SON from top to bottom respectively) between ssp3-7.0 over the period 2071–2100 and historical mean state (1985–2014) for (**a**) 2 m daily relative humidity, (**b**) cumulated precipitation and (**c**) surface mean daily wind speed.

Seasonal detailed differences maps are provided for SSP1-2.6 and SSP3-7.0 in Figs. 12, 13 respectively, considering the same projection (2071–2100) and reference (1985–2014) period used in previous analysis (Figs. 10, 11). A general increase in temperature (mean, maximum and minimum; Fig. 12a–c respectively) is shown for each season (DJF: from December to February; MAM: from March to May; JJA: from June to August; SON: from September to November) with values reaching up to +2.8 °C for each season, except JJA, which experiences higher differences between the future and past climate up to ~4.3 °C. Very localised areas of Sardinia, Calabria and Sicily may be interested in higher differences (up to ~3.8 °C) in MAM for daily mean temperatures. In contrast, as also observed over the entire annual based estimates (Figs. 10,11), only small areas over Italy experience a decrease in surface daily wind speed (between $-0.1\div-0.2$ m/s). In particular, those differences are more pronounced over southern regions, especially during the fall season (Fig. 12d). The accumulated precipitation (Fig. 12e) presents an inter-seasonal oscillation in different patterns: during DJF, most of Italy, except the southernmost regions and the islands, experience positive differences (~+1 mm) associated with higher accumulated daily precipitation, while a dramatic shift toward a drier regime is observed in spring (MAM). Here, only the northernmost sector still maintains a negative pattern, while most of Italy may be affected by a general decrease in precipitation (~−1mm). This pattern becomes more pronounced in summer (JJA), restricting the negative differences to an even more localised northeast area. These differences are minimised during fall (SON), with no significant values over most of Italy, except negative values over the Campania, Calabria, Sicily and Sardinia regions that contrast positive ones over Friuli Venezia Giulia and spotter regions of Liguria, Lombardia and Abruzzo region. As observed over the annual-based analysis (Fig. 11a), where the mean relative humidity is characterised by negative differences over the entire Italy, this pattern is expected in each season. In particular, the driest one may be summer with values decreasing down to ~−16% (Fig. 12f).

For scenario SSP3-7.0 differences observed in SSP1-2.6 are stronger (considered as absolute values) as expected. Differences up to ~9.4 °C are reached in summer both for mean, maximum and minimum temperatures (Fig. 13a–c), with stronger peaks over southern Italy, Sicily, Sardinia and Emilia Romagna. Then, high differences are expected in fall with peaks up to ~6.6 °C in southern Italy, while during winter and spring, higher temperatures are captured, reaching values up to ~5.7 °C. Daily mean wind speed in SSP3-7.0 (Fig. 13d), with respect to SSP1-2.6, presents higher negative differences over most of Italy (~−0.5 m/s), with peaks found in winter over the Emilia Romagna, Campania and Apulia region in fall and winter, while the areas more affected by the largest reduction in spring are Emilia Romagna and Apulia region. Finally, in summer the Apulia region may be hit by stronger differences (~−0.5 m/s). The precipitation patterns captured by SSP3-7.0 differ significantly with respect to SSP1-2.6 for summer and fall seasons, where the entire Italy, except few localised northernmost areas, are interested by negative patterns. Also, during winter and spring, with respect to scenario SSP1-2.6, wider regions are interested by negative values. Similar patterns as those shown in Fig. 12f, are observed in the mean daily relative humidity under SSP3-7.0 conditions (Fig. 13f). No significant differences between SSP1-2.6 and SSP3-7.0 are captured in winter, while a clear shift toward less humid conditions is observed for spring, summer and fall, with differences maximized in JJA (~−16%). By visual inspection, areas with higher peaks may be northwestern and central Italy.

**Fig. 14:**
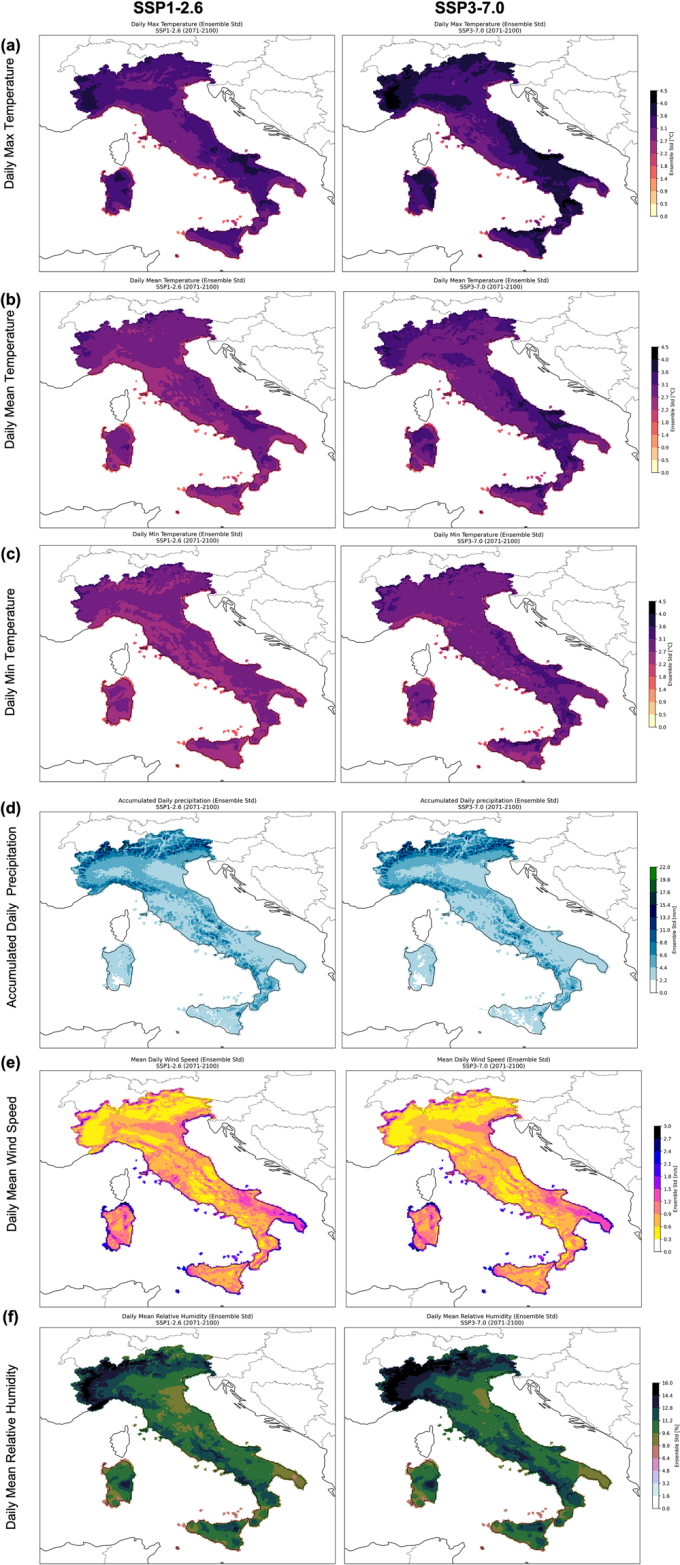
Ensemble standard deviation map under the ssp1-2.6 (center) and ssp3-7.0 (right) scenarios for 2 m (**a**) maximum, (**b**) mean, (**c**) minimum daily temperature, (**d**) cumulated daily precipitation, (**e**) surface daily mean wind speed and (**e**) 2 m mean daily relative humidity and (**e**) cumulated daily precipitation.

Finally, since climate projections by construction are affected by uncertainty, ensemble standard deviation maps are provided in Fig. 14 to support ensemble mean results discussed above. Maps for each ECV and climate projection are provided to emphasise which are the regions affected by higher uncertainty, based on the adjusted ensemble GCMs here considered. For maximum daily temperature (Fig. 14a), under SSP1-2.6 condition, the regions with higher standard deviation (~4.5 °C) are Piemonte and Apulia regions, surrounded by values between ~1.8 $\div$ 3.6 °C. Moreover, a relevant uncertainty source comes from the scenarios condition. In fact, the ensemble spread over *tasmax* strongly increases in scenario SSP3-7.0, doubling the standard deviations in some areas. Standard deviations over daily mean temperature values (Fig. 14b), reach values up to ~3.6 °C in SSP1-2.6 and ~4 °C in SSP3-7.0, maintaining similar patterns between the two scenarios, with spread slightly higher in a few areas over the SSP3-7.0 scenario. The minimum daily temperature with respect to the previous variables, looks affected by lower standard deviation for both scenarios (Fig. 14c).

Unlike what was observed for temperature, the patterns of standard deviation in the two scenarios are very similar and strongly driven by orography. High standard deviation values are found in the Alps, in some Apennine areas, and along the southwestern coasts. Maximum values reach up to ~22 mm in confined areas of the Alps. In small areas of south-central Sardinia and Sicily, the standard deviation is negligible, indicating strong model coherence (Fig. 14d). As observed for precipitation, the standard deviation of daily mean wind speed is also very similar between the two scenarios (Fig. 14e). Maximum uncertainty values (around 3 m/s) are found in Apulia, Sardinia, along the coasts, and in very confined areas of the Alps. As observed for precipitation and wind, the spatial distribution of uncertainty for humidity is also very similar between the two scenarios. The highest values occur in the northwest and extend throughout the Alpine arc, reaching up to approximately 16%. Elevated values are also found in the central Apennines, along the Salernitan and Calabrian coasts, and in some areas of northern Sicily and central-eastern Sardinia.

The results demonstrate that this dataset well captures the observed variability over the training period, minimizing biases while providing detailed, local-scale insights into future climate and its associated uncertainty. By offering valuable information on the spatial distribution of uncertainty and ensemble mean expected values, it becomes an essential tool for impact studies aimed at identifying highly accurate mitigation and adaptation measures. Consequently, despite the limitations discussed earlier, stemming from the chosen bias-correction method and reference dataset, SD-EQM_GCMs_IT^30^ successfully meets the scientific community’s needs.

## Data Availability

Data are available on the CMCC Data Delivery System (DDS) at the link: https://dds.cmcc.it/#/dataset/cmip6-stat-downscaled-over-italy/. While annexes may be visualized and downloaded on Zenodo (10.5281/zenodo.15048021)^37^. Moreover, climate indicator maps derived by using this dataset are provided on the CMCC Dataclime platform (https://www.cmccwebremhi.it/indicatorcmip6). The code used for applying Empirical Quantile Mapping to the CMIP6 data is publicly available on the Github: https://github.com/FedeleG/Bias-correction. The CMCC Foundation ensures its data undergoes through verification processes. However, it does not accept liability for inaccuracies or omissions. Furthermore, the CMCC Foundation is not responsible for data interpretations, modifications, or content produced by third parties, nor for any materials or conclusions derived from external sites based on this data. Users bear full responsibility for any decisions made using this data, and the CMCC Foundation disclaims liability for any direct or indirect losses or damages resulting from its use.
